# Supplementary material for: Energy, Sodium, Sugar and Saturated Fat Content of New Zealand Fast-Food Products and Meal Combos in 2020
Source: Nutrients. 2021 Nov 10;13(11):4010. doi: 10.3390/nu13114010 (PMC8625722; doi:10.3390/nu13114010)
Supplement: Supplementary file 1 [file nutrients-13-04010-s001.zip › nutrients-1433297-supplementary.pdf]

Supplementary Table S1. NZ fast food supply 2020, by food category: Minimum and maximum energy, sodium, total sugar and saturated fat content per serving and percentage contribution to recommended daily intakes of energy, sodium, sugar and saturated fat.

Major and minor fast food category	N	Energy- Kilojoules/serving		Sodium- milligrams/ serving		Total sugar-grams/serving		Saturated fat-grams/serving	
		Content	Percentage of daily recommendation*	Content	Percentage of daily recommendation*	Content	Percentage of daily recommendation*	Content	Percentage of daily recommendation*
		Min-Max	Min-Max	Min-Max	Min-Max	Min-Max	Min-Max	Min-Max	Min-Max
Asian	48	789.6 - 5,170.0	9.1 - 59.4	352.0 - 2,060.0	9.0 – 103.0	0.1 - 28.1	0.2 – 55.1	0.3 - 10.1	1.3 – 43.9
Beverages									
Milkshakes, smoothies	130	340.0 - 5,300.0	3.9 - 60.9	2.0 - 1,040	0.1 – 52.0	8.7 - 199.0	17.1 – 390.2	0.0 - 41.6	0.0 – 180.9
Soft Drinks, sugar sweetened	72	193.0 - 1,390.0	2.2 - 16.0	0.3 - 390.0	0.0 – 19.5	7.3 - 72.0	14.3 – 141.2	0.0 - 4.2	0.0 – 18.3
Soft Drinks, artificially sweetened	24	3.0 - 139.00	0.0 - 1.6	0.0 - 310.0	0.0 – 15.5	0.0 - 0.3	0.0 – 0.6	0.0 - 3.3	0.0 – 14.4
Breakfast, savoury	36	571.0 - 3,300.0	6.6 - 37.9	11.0 - 2,080.0	0.6 – 178.7	0.3 - 36.6	0.6 – 71.8	0.7 - 27.2	3.0 – 221.7
Pastry, savoury	88	506.0 - 2,639.0	5.8 - 30.3	33.3 - 1,850.0	1.7 – 92.5	0.2 - 12.2	0.4 – 23.9	2.2 - 23.0	9.6 – 109.4
Cakes, muffins and pastry	315	436.0 - 7,079.0	5.0 - 81.4	3.0 - 1,600.0	0.2 – 80.0	3.1 - 183.0	3.9 – 358.8	0.5 - 43.0	1.9 – 187.0
Desserts	75	189.0 - 10,099.0	2.2 - 116.1	4.0 - 1,504.0	0.2 – 75.2	4.7 - 302.0	9.2 – 592.2	0.1 - 36.6	0.0 – 159.1
Burgers	149	1,080.0 - 8,970.0	12.4 - 103.1	415.0 - 3,069.4	19.8 – 172.0	2.6 - 39.3	5.1 – 77.1	1.7 - 73.6	4.4 – 320.0
Chicken	63	57.0 - 4,960.0	0.7 - 57.0	47.0 - 3,040.0	2.4 – 204.1	0.0 - 13.3	0.0 – 53.0	0.1 - 21.2	0.4 – 138.0
Pizza	416	451.0 - 2,972.0	5.2 - 34.2	133.0 - 1,306.0	6.7 – 159.9	0.7 - 15.8	1.4 – 58.8	0.2 - 13.8	0.8 – 127.8
Salads	59	70.0 - 2,750.0	0.8 - 31.6	6.0 - 1,950.0	0.2 – 168.5	0.6 - 31.6	1.2 – 66.9	0.0 - 15.0	0.0 – 108.5
Sandwiches and wraps	113	702.0 - 3,550.0	8.1 - 40.8	165.0 - 2,460.0	8.0 – 222.5	0.7 - 15.9	1.4 – 51.6	0.4 - 25.9	0.9 – 117.4
Fries	25	806.0 - 6,350	9.3 - 73.0	101.0 - 2,562.0	5.1 – 128.8	0.0 - 62.4	0.0 – 122.4	1.0 - 14.0	4.4 – 60.9
Sides, other	52	7.0 - 5,724.0	0.1 - 65.8	1.0 - 3,218.4	0.1 – 160.9	0.0 - 59.4	0.0 – 116.5	0.0 - 44.6	0.0 – 193.9
Dressings/condiments, savoury	88	23.0 - 955.0	0.3 - 11.0	23.5 - 974.0	0.6 – 48.7	0.0 - 15.3	0.0 – 30.0	0.0 - 7.4	0.0 – 32.2

*Percentage calculated having as reference the recommended adult average daily energy intake (8700 kilojoules/day), sodium intake (2000 mg/day), free sugars intake (maximum 51g/day based on 8700kJ), saturated fat intake (maximum of 23g/day based on 8700kJ). **Missing (n) Nutrient content and % of daily recommendation: Asian** (11); Milkshakes, smoothies (0); Soft drinks-sugar sweetened (1); Soft drinks-artificially sweetened (0); Breakfast-savoury (0); Pastry-savoury (0); Cakes, muffins and pastry (0); Desserts (0); Burgers (0); Chicken (6); Pizza (1); Salads (0); Sandwiches and wraps (0); Fries (0); Sides, other (0); Dressings/condiments-savoury (0)

Supplementary Table S2. NZ fast food supply 2020, by meal combo: Minimum and maximum energy, sodium, total sugar and saturated fat content per serving and percentage contribution to recommended daily intakes of energy, sodium, sugar and saturated fat.

Types of fast food combos	N	Energy- Kilojoules/serving		Sodium- milligrams/ serving		Total sugar- grams/serving		Saturated fat- grams/serving	
		Content	Percentage of daily recommendation*	Content	Percentage of daily recommendation*	Content	Percentage of daily recommendation*	Content	Percentage of daily recommendation*
		Min-Max	Min-Max	Min-Max	Min-Max	Min-Max	Min-Max	Min-Max	Min-Max
Burger(s), fries, drink-SSB	20	2,809.0 – 6,434.0	32.3 – 74.0	652.1 – 2,794.0	32.6 – 139.7	28.4 – 71.1	55.7 – 139.4	3.2 – 28.1	13.9 – 122.2
Burger(s), fries, drink-ASB	20	2,330.0 – 5,666.0	26.8 – 65.1	679.0 – 2,816.5	34.0 – 140.8	4.1 – 16.3	8.0 – 32.0	3.2 – 67.9	13.9 – 295.2
Burger(s), fries, dessert, drink-SSB	9	5,421.0 – 8,609.0	62.3 – 98.95	1,517.0 – 2,630.0	75.9 – 131.5	65.0 – 91.8	127.5 – 180.0	8.5 – 50.2	37.0 – 218.3
Burger(s), fries, dessert, drink-ASB	9	4,653.0 – 7,841.0	53.5 – 90.1	1,537.0 – 2,650.0	76.9 – 132.5	30.9 – 46.2	60.6 – 90.6	8.5 – 50.2	37.0 – 218.3
Chicken, fries, drink-SSB	3	3,343.0 – 4,858.0	38.4 – 55.84	1,353.0 – 1,996.5	67.7 – 99.8	39.1 – 52.5	76.7 – 102.9	3.4 – 9.2	14.8 – 40.0
Chicken, fries, drink-ASB	3	2,539.8 – 4,189.2	29.2 – 48.2	1,375.5 – 2052.8	68.8 – 102.6	0.1 – 4.8	0.2 – 9.4	3.4 – 9.2	14.8 – 40.0
Chicken, fries or potato, dessert, drink-SSB	10	2,980.0 – 8,193.0	34.3 – 94.2	1171.0 – 3068.0	58.6 – 153.4	41.0 – 66.9	80.4 – 131.2	6.2 – 29.6	27.0 – 128.7
Chicken, fries or potato, dessert, drink-ASB	10	2,395.0 – 7,425.0	27.5 – 85.3	1,174.0 – 3,088.0	58.7 – 154.4	6.0 – 21.3	11.8 – 41.8	6.2 – 29.6	27.0 – 128.7
Sandwich, fries, drink-SSB	6	3,249 – 4,202.9	37.3 – 48.3	771.0 – 1,610.6	38.6 – 80.5	49.6 – 55.4	97.3 – 108.6	2.8 – 8.5	12.2 – 37.0
Sandwich, fries, drink-ASB	6	2,445.8 – 3,399.7	28.1 – 39.1	793.5 – 1,633.1	39.7 – 81.66	1.9 – 7.7	3.7 – 15.1	2.8 – 8.5	12.2 – 37.0
Pizza(s), Side(s)	13	2,505.6 – 4,138.0	28.8 – 47.6	728.0 – 1,750.0	36.4 – 87.5	8.9 – 50.8	17.5 – 99.6	8.0 – 16.9	34.8 – 73.5
Pizza(s), Side(s), Drink-SSB	5	4,249.0 – 4,765.0	48.8 – 54.8	711.0 – 1,272.0	35.6 – 63.6	71.9 – 84.5	141.0 – 165.7	10.5 – 16.9	45.7 – 73.5
Pizza(s), Side(s), Drink-ASB	5	3,659.6 – 4,175.6	42.0 – 48.0	714.3 – 1,275.3	35.7 – 63.8	36.9 – 49.5)	72.4 – 97.1	10.5 – 16.9	45.7 – 73.5

Pie, side (optional), drink-SSB	4	3,697.0 – 4,627.0	42.5 – 53.2	881.0 – 1,400.0	44.1 – 70.0	47.4 – 67.1	92.9 – 131.6	8.0 – 31.2	34.8 – 135.7
Pie, side (optional), drink-ASB	4	2,946.0 – 3,876.0	33.9 – 44.6	902.0 – 1,421.0	45.1 - 71.1	2.4 – 22.1	4.7 – 43.3	8.0 – 31.2	34.8 – 135.7
Breakfast	3	4,051.0 – 4,861.0	46.6 – 55.9	1,993.0 – 2,422.0	99.7 – 121.1	28.6 – 30.6	56.1 – 60.0	10.9 – 20.1	47.4 – 87.4
Salad or wrap, smoothie	3	1,800.0 – 4,539.0	20.7 – 52.2	362.0 – 1,021.0	18.1 – 51.1	47.2 – 56.7	92.6 – 111.2	1.8 – 15.5	7.8 – 67.4
Burger or chicken and fries	3	2,422.0 – 3,488.2	27.8 – 40.1	1,125.0 – 1,566.0	56.3 – 78.3	0.0 – 7.3	0.0 – 14.3	6.8 – 9.7	29.6 – 42.2
Chicken, potato, fries, additional item**, drink-SSB	20	4,971.1 – 7,774.1	57.1 – 89.4	2,194.0 – 3,936.0	109.7 – 196.8	57.3 – 101.2	112.4 – 198.4	6.2 – 17.0	27.0 – 73.9
Chicken, potato, fries, additional item***, drink-ASB	20	4,167.9 – 6,970.9	47.9 – 80.1	2,216.5 – 3,958.5	110.8 – 197.9	9.6 – 53.5	18.8 – 104.9	6.2 – 17.0	27.0 – 73.9
**Total**	**176**	**1,800-8,609.00**	**20.7-98.95**	**362.0-3,958.5**	18.1-197.9	**0.0-101.2**	**0.0 – 198.4**	**1.8-67.9**	**7.8 – 295.2**

SSB: sugar sweetened beverage, ASB: artificially sweetened beverage

*Percentage calculated having as reference the recommended adult average daily energy intake (8700 kilojoules/day), sodium intake (2000 mg/day), free sugars intake (maximum 51g/day based on 8700kJ), saturated fat intake (maximum of 23g/day based on 8700kJ).

** Burger, sandwich, bread roll or coleslaw.
